# Remarks on Scepticism, Especially as It Is Connected with the Subjects of Organization and Life. Being an Answer to the Views of M. Bichat, Sir T. C. Morgan, and Mr. Lawrence, upon Those Points

**Published:** 1819-07-01

**Authors:** 


### Medico-Chirurgical Journal;

OR,

### Quarterly Register

OF

### MEDICAL AND SURGICAL SCIENCE.

No. 5.] JULY 1, 1819. [Vol. II.

### First Department.

### BIBLIOLOGY;

OR,

ANALYTICAL REVIEWS OF MEDICAL PUBLICATIONS,

*Interwoven with*

ORIGINAL OBSERVATIONS, AND PRACTICAL RESEARCHES.

Nec tibi quid liceat sed quid fecisse decebit
Occurrat, mentemque domat respectus honesti.

Claud.

### I.

*Remarks on Scepticism, especially as it is connected with the Subjects of* Organization and Life. *Being an Answer to the Views of M. Bichat, Sir T. C. Morgan, and Mr. Lawrence, upon those Points.* By the Rev. Thomas Rennel, A. M. &c. *Octavo, pp.* 140. *London,* 1819.

If it be true that Happiness is our being's end and aim,

That something which still prompts th' eternal sigh,
For which we bear to live, or dare to die,

Then the subject of the volume before us is one of vital importance to society at large, but particularly to the medical profession, at this moment. It is in vain to deny that a well grounded suspicion has gone abroad, among the community in general, that the doctrine of materialism has taken deep root in the medical world. This suspicion, or rather conviction, is calculated to prove highly injurious in two ways—First, it must injure the profession itself in the eyes of those who are capable of distinguishing sophistry from truth, and assumptions from facts. Secondly, the plea of a learned and enlightened body having adopted such tenets as those of materialism, must prove an imposing excuse for the profligate and unprincipled part of society embracing and inculcating them. The same reasons will decide the wavering and unsettled creeds of a considerable proportion of those in the lower walks of life, who are incapable of analysing the doctrines of these "modern philosophers," and who will consequently fall into the tenets of the new light, on the faith of their superiors.

For several years past we have observed, with concern, a focus of these Gallic doctrines and principles, in the midst of a medical school of eminence in this metropolis; whence emanated, in all directions, through the medium of Cyclopœdia's, Lectures, and Demonstrations, the tenets of materialism! We were often astonished, and we publickly expressed that astonishment, in the monthly series of this Journal, that the wise and good portion of society at large did not rise, en masse, to shame and discountenance those vehicles of such pernicious principles, and thus check the dissemination of a fatal poison through the veins of the community at large. Many of the Medical articles in Rees's Cyclopœdia, which are literally translated from the French, without acknowledgment, are calculated to do infinitely more mischief than Tom Paine's Age of Reason, being the more dangerous as they are conveyed in the garb of truth, and mixed up with accurate and enlightened views of Physiology. Well may Mr. Rennel say,

"The Treatises which I have been induced to notice, strike deep at the root of all religion, both natural and revealed; and, as I have been lately informed by the best authority, upon the minds of many they have had a considerable effect."

It is lamentable indeed to think, that the members of our profession, who are constantly contemplating the wonderful wisdom of the Creator, as displayed in the construction of the human frame, should be the foremost, among modern philosophers, to level man with the grass he treads on; to deny the possibility of a future state of existence; to laugh at all ideas of moral responsibility after death, and thus to break down every barrier against crime but the axe and the gibbet! We would ask our materialist brethren, whether they believe (granting, for a moment, that they alone can unveil the secrets of the grave) that society is yet arrived at that degree of perfection, which renders all Divine laws quite unnecessary? And, granting still farther, that human laws were equal to the restraint or punishment of vice, is it charitable, is it humane, to not only withdraw all incentives to virtue, but to tear away every prop and consolation from those who are suffering under the pangs of irremediable disease—the oppressor's wrong—or the storms of unmerited adversity?—The medical philosopher, in truth, has no occasion to go out of his own daily walk for opportunities of observing the benign influence of Religion on those whose bodies are broken down with pain, or whose minds are tortured with anxiety; and we cannot but suspect, that the man who can deliberately subvert these consolations of human misery, is himself deficient in humanity towards his fellow creatures, and totally insensible to their feelings, their hopes, and their fears.

And this reflection leads to the work before us, in which Mr. Rennel has very accurately traced scepticism to its principal causes, moral and intellectual. In the first class he justly considers "*the indulgence of licentious habits*," as an operating one of primary consequence. Scepticism is most indulgent to the passions; and this indulgence is the uniform end and determination of all its objections, and all its principles. Whether it be liberty, or whether it be necessity, which it would inculcate—*all responsibility for action is to be abolished.* "Sceptics will make the soul a component part of this frail and corruptible frame, that it may perish with the organ and instrument of its lusts." Among the few sceptics, "whose morals are coldly correct, there are other causes which supply the place of licentious indulgences, as pride, &c."

In respect to our own profession, there certainly appears to be something in its study and practice, which hardens the mind against those impressions of religious awe, that stamp themselves with such force on the minds of others. The spectacles of mortality, which the medical man daily witnesses, lose their terrific character by repetition, while the power which he posseses over the human frame, and his constant contemplation of *secondary causes,* too often tend to draw his attention from the FIRST GREAT CAUSE of all things. But how much more must this natural tendency be increased, if the mind of the youthful student be early poisoned by the doctrines of Materialism and Atheism, now publicly taught in a distinguished School of the British metropolis? Surely, the men who thus, in defiance of every religious and virtuous sentiment, brave the general indignation of Society at large, as well as the feelings of the parents and guardians of medical youths, must have hearts of marble, and nerves of iron!

"If there be a thought which, in the hour of impending dissolution, must agonize and distract even the most hardened infidel, it is the remembrance of having been the instrument of perplexing the understandings, destroying the hopes, and corrupting the morals of the young men committed to his charge. At that very age when every motive which Religion can supply, is imperiously called for, to check the rising passions, and to subdue them into a state of rational and permanent restraint, it is an offence no less against social than individual happiness, to inculcate those principles which set all conscience and morality at defiance. The man who will coldly and laboriously teach the lessons of infidelity, will not scruple to excuse, if not to inculcate the practice of immorality; and he who will confound the distinctions between truth and falsehood in speculation, will annihilate the boundaries between virtue and vice in practice. Nor will the mischief stop here, nor confine itself to those who have been the more immediate victims of his delusion. Infidelity, like every other pestilence, is propagated by contagion. In whatever provincial town these young men may settle, they will find but too many of their own rank and age, who will become ready converts to a principle which, while it flatters their understanding, corrupts and indulges their heart." 52.

This is a picture which it behoves the parents and guardians of the rising generation of the Profession to seriously contemplate.

"I am at a loss, says this excellent Divine, to imagine what worthy end, or even what plausible excuse, a teacher can propose to himself for the propagation of opinions which unsettle and distract the mind, destroy every good and moral feeling, and deprive their victim of all comfort in the day of affliction; of all hope on the bed of death. Will either the principles or the practices of the gospel render the student less ardent in the pursuit of knowledge, or less active in the duties of his profession? Will it exclude any one light of philosophy, any one ray of science from his mind? Will it make him less tender in his manners, less kind in his actions, especially to the poor and the friendless? Will it not rather give him a power over the mind as well as over the body of his patient; so that while he relieves the sufferings of the outward frame, he may speak in the language of peace and of comfort to the soul?" p. 52.

But to come more to our immediate purpose. In the 5th Chapter of the work before us, Mr. Rennel has very ably combated "the views of M. Bichat, of Sir T. C. Morgan, and of Mr. Lawrence," respecting Life and Organization. It is the aim of these physiologists to impress upon the mind an erroneous notion of LIFE, and to represent it as entirely the result of organization. According to the first of these gentlemen, the *passions* are organic impulses, and organic life the assemblage of those functions which the animal has in common with the vegetable. Thus a cabbage and a man, having the functions of organic life in common, and the passions being among those functions, it follows that jealousy, anger, revenge and love, are the common affections of the man and of the cabbage!

Sir T. C. Morgan, fresh from the Parisian school of atheism, brings out the following learned definition of life. "*The sum total of functions, which any individual can perform, constitutes its life.*" Thus, then, life is not the cause, but the *effect!* Life is the sum total of secretion, locomotion, volition, and other functions which any individual is able to perform;—but in what consists the *ability* of performance, the author has not informed us. "Good and evil, says he, are principles intelligible only as they relate to the laws of *organic* existence!" Excellent morality! Thus the forging of a bank note is an evil intelligible only, as it may lead to a *stoppage of respiration* at the Old Bailey!

All our ideas of reflection are produced, according to this author, by external impact. "As far therefore, at least, as ideas are the subjects of human contemplation, they must be regarded as changes impressed upon the substance of the brain, by the impact of bodies that are external to its tissue." 260.

The vibrations of Hartley, as Mr. Rennel well observes, were but powerless instruments compared with the *impact* of this new philosopher.

"An *unlucky* blow, says the Cambrian knight, inflicted during a paroxysm of rage, is more likely to *subdue* a tendency to that passion, than the best arguments which reason can suggest." Thus, according to the new doctrines of organic impulses, it is not to the feeling of shame, of humiliation, or of reflection, which either the blow or the pain which it causes, may excite, that its beneficial effects are to be ascribed — No; — it is the *impact* on the skull which subdues the tendency of every bad and rebellious passion! From these specimens of our author's ratiocination, the reader will probably apprehend little danger from the principles broached in the "Philosophy of Life," though copied from the school of Bichat, Cabanis, and other worthies of the continent. He modestly enough acknowledges that "Scylla and Charybdis have yawned upon his track, and that he has probably been often immerged in *both* vortices."

In respect to Mr. Lawrence's introductory lectures, they are of a still more dangerous tendency, from the high ground, as a surgeon and anatomist, on which this gentleman stands, and consequently from the avidity with which his principles and doctrines will be imbibed by the numerous students of the Bartholomew school. In this work Mr. Lawrence has plainly told his hearers that *medullary matter* is capable of sensation and *thought;* that there is no independent living principle superadded to the structure of animal bodies; that life is the result of organization. Surely Mr. Lawrence must be aware that these principles lead to the very worst conclusions. Materialism and atheism go hand in hand; for, when once we have argued ourselves out of the existence of our soul, which is a spirit, by the very same process we argue ourselves out of the existence of the Almighty, who is a spirit also. Mr. Rennel has criticized Mr. Lawrence's doctrines with such acuteness and force, that we shall introduce a page or two of Mr R's work in this place.

The following is the view of the subject which Mr. Lawrence has taken.

"Organization means the peculiar composition which distinguishes living bodies; in this point of view they are contrasted with inorganic, inert, or dead bodies. Vital properties, such as sensibility, irritability, are the means by which organization is capable of executing its powers; the vital properties of living bodies correspond to the physical properties of inorganic bodies; such as cohesion, elasticity, &c. Functions are the purposes which any organ or system of organs executes in the animal frame; there is of course, nothing corresponding to them in inorganic matter. Life is the assemblage of all the functions, and the general *result* of their exercise. Thus organization, vital properties, functions, and life, are expressions related to each other, in which organization is the *instrument*, vital properties the acting power, function the mode of action, and life the result."

So then, we have an instrument, an acting power, a mode of action, and a result. All this is very intelligible. Organization then is the instrument which produces life as its result. But in the first sentence Mr. Lawrence informs us, that organization "is the peculiar composition which distinguishes living bodies, as contrasted with inorganic or dead bodies." Here then, it appears, that life so far from being the "result," is in fact, a "component part" of the said instrument; and that so far from life being the consequence or result of organization, no organization can exist without it! So, according to Mr. Lawrence, "life is the result of the peculiar composition which distinguishes living bodies"; or, in other words, we first take for granted the existence of life, and then we prove it to result from its own existence! This is "Medical Logic" with a vengeance!

"Life," again, says Mr. Lawrence, is the *assemblage* of all the functions, and general *result* of their exercise." Just now he made the result coexisting with the instrument of its production, and now he makes it the same with the mode of action; or, in other words, with the mode of producing it!

Let us take Mr. Lawrence upon his own ground. A scalpel is the instrument, a hand the acting power, cutting the mode of action, and a wound the result. What would Mr. Lawrence say to the man who should assert, that the wound was coexistent with the scalpel; or again, that the act of cutting was a wound?

After all this, in the very next page, Mr. Lawrence informs us, that *the vital properties or forces animate living matter, so long as it continues alive;* or, in other words, that they animate [give life] to matter which *has* life, so long as it continues to *have* life!

First, then, we were told that organization was the instrument, and life the result; we were next told, that organization and life were coexistent; and now we are told,

> "The result of all these inquiries I have no hesitation in affirming to be, that no connection has been established, in any one case, between the organic texture and its vital power."

To such palpable absurdities, as Mr. Rennel justly observes, are men of the highest professional eminence reduced, when they would annihilate that first, that noblest gift of God to man—the IMMORTAL SOUL.

The plain fact is, that when we would account for *modes* of operation, and investigate the nature of *primary* causes themselves, we go beyond the reach of our faculties, and all is mystery and confusion. And when we acknowledge as we must, our ignorance of almost all the more common phenomena of Nature around us, is it not culpable presumption to issue forth dogmatical doctrines that strike at the root of natural and revealed religion; of social and political happiness? Doctrines that take away all stimulus to virtue; all hopes of future reward; all idea of moral responsibility; and, consequently, all fear of future punishment!

Here we shall conclude, by recommending to the careful perusal of every one who wishes well to the cause of religion and virtue, the little volume of the Rev. Mr. Rennell. It evinces great physiological acuteness, and exhibits one of the finest specimens of dignified philosophical reasoning, which we have read since the days of Paley. No man, who has the superintendance of medical youth, should fail to put it into the hands of his pupils, and inculcate its views and principles, as he hopes for their welfare in this world, and happiness in the next.

